# The Organizational Atmosphere in Israeli Hospital during COVID-19: Concerns, Perceptions, and Burnout

**DOI:** 10.3390/ijerph18115544

**Published:** 2021-05-22

**Authors:** Osnat Bashkin, Nadav Davidovitch, Noam Asna, Doron Schwartz, Keren Dopelt

**Affiliations:** 1 Department of Public Health, Ashkelon Academic College, Ashkelon 78211, Israel; dopelt@bgu.ac.il; 2 Department of Health Systems Management, Ben-Gurion University of the Negev, Beer Sheva 84105, Israel; nadavd@bgu.ac.il; 3 Ziv Medical Center, Oncology Institute, Safed 13100, Israel; noama@ziv.gov.il; 4 Risk Management Unit, Soroka University Medical Center, Beer Sheva 84101, Israel; doronsh@clalit.org.il

**Keywords:** COVID-19, coronavirus wards, concerns, perceptions, burnout, healthcare systems, organizational culture, management

## Abstract

The COVID-19 crisis poses challenges to healthcare systems and requires micro- and macro-organizational adaptations. This study examined the organizational atmosphere in Israeli hospitals by evaluating workers’ perceptions and concerns about the COVID-19 crisis and its management. At the end of the pandemic’s first wave in Israel, 547 healthcare workers responded to an online survey, which inquired about COVID-19 concerns at the individual and family level, perceptions at the national and organizational level, perceptions of the way the crisis was managed, self-assessment of coping with the crisis and burnout, and demographics. Findings showed that healthcare workers expressed deep concerns for family members and apprehension at a national level. Respondents noted that they were coping well with the crisis while expressing negative perceptions of how the crisis was managed. A regression model showed that the low self-assessment of medical staff of coping with the crisis, deep concerns at the organizational level, negative perceptions of crisis management, and providing care for COVID-19 patients were predictors of burnout. The findings emphasize the importance of developing a supportive organizational culture for hospital workers. Awareness of their concerns and perceptions is essential to improve organizational culture and healthcare systems’ ability to continue fighting the virus and confront future health crises.

## 1. Introduction

The COVID-19 crisis that has burdened the world for the past year posed a significant challenge to healthcare systems and their employees. Aside from the critical issue of managing patient care and strategies to prevent the spread of the pandemic, healthcare systems were forced to make organizational adjustments on all levels to deal with the crisis. Shifts were extended from eight to twelve hours, employees underwent special training, and the nature of routine work changed; at the logistical level, existing wards were converted to COVID-19 wards; at the organizational culture level, new procedures for receiving patients were implemented, and new policies for protection and hygiene were introduced; patient accompaniment and visitation rules changed, etc. In managing an ongoing crisis, organizational adaptations are necessary to protect medical personnel from infection as well as from physical and emotional burnout [1]. Medical staff are at the front line of the battle against COVID-19. They must cope with a crisis with personal health and occupational ramifications, implement variegated organizational changes, and cope with ongoing job stressors, which may lead to adverse mental health symptoms and may affect the quality of medical care provided to patients [2].

Recent studies showed that medical staff expressed high levels of concern related to a range of aspects of COVID-19. In a recent survey conducted in China, medical personnel expressed deep concerns regarding the effectiveness of measures to protect them against infections, and for medical colleagues without protective equipment. Most of them also expressed worries of infecting family members [3]. In another recent study, 1795 medical staff from Taiwan responded to a survey, with most of them working in emergency units treating COVID-19 patients, and some of the respondents had previously experienced pandemic outbreaks such as SARS and MERS. Almost half of the respondents expressed feelings of burnout and most of them expressed high anxiety levels (78%) [4]. An assessment of mental health symptoms among healthcare workers who had direct contact with COVID-19 patients showed a significantly higher risk of mental symptoms such as anxiety, depression, and sleep disorders compared to second line workers not directly involved with taking care of COVID-19 patients [5]. In a similar latest study in Turkey, medical staff noted that the COVID-19 outbreak has implications for their mental health and expressed high rates of emotional exhaustion [6]. In line with recent studies, a past study on earlier pandemic outbreaks, such as the SARS and the MERS pandemics, suggested that global crises significantly affect healthcare workers by creating job stressors that cause strain symptoms [7].

In times of global health crisis, common work stressors include high physical and mental workloads, hazardous work environments, uncertainty in work instructions, ambiguous infection control guidelines, and rapidly changing policies [8]. Prolonged exposure to emotional and interpersonal stressors in the workplace is the leading cause of burnout [9]. According to the demand/resources model of stress (JD-R model) [10], a feeling of burnout may develop due to an increase in work demands, leading to exhaustion, and, alongside limited resources, lead to low work engagement. Work demands and resource availability are part of the organizational culture of a workplace. Furthermore, organizational culture aspects may prevent burnout, such as teamwork, managerial support, autonomy in decision making, and adequate working conditions [11]. A study that examined the relationship between burnout, work demands, and organizational culture among nurses in a hospital in Macedonia found a positive correlation between work demands at the organizational level and burnout and a negative correlation between work demands and work engagement. A positive correlation was found among physicians between work demands on the emotional level and burnout [12].

In the current challenging times of the global COVID-19 crisis, hospital workers must cope with unique work demands on both an organizational and emotional level. Increasing work demands, time pressure, a chaotic work environment, and critical perceptions of the organizational culture have been found to correlate with burnout, decreased satisfaction, stress, and intentions to quit among physicians [13]. However, a recent study in China that examined burnout among medical staff during the pandemic found lower reports of burnout among medical staff who worked directly with COVID-19 patients than medical staff who continued working on their routine wards [14]. It seems that working at the front lines of the pandemic engenders a sense of involvement and control in unprecedented circumstances, which increases the sense of commitment and reduces the prevalence of burnout [15]. Recent research’s findings thus far show widespread fear of infection among healthcare workers, leading to higher levels of stress [16]. Routine work becomes more stressful when resources for patient care are insufficient in the context of high infection rates [17]. Likewise, negative perceptions of workers’ organizational support during the pandemic were linked to stress symptoms and concern for personal and family health [18]. The increased demands on healthcare workers during a health crisis may exceed their abilities to cope with the ongoing crisis and lead to anxiety, stress, and burnout [19]. Thus, it is crucial to examine healthcare workers’ feelings and perceptions during the pandemic, improve health systems’ organizational preparedness, and the ability to continue the fight against the current pandemic and future health crises that we may encounter. This study aims to examine organizational and managerial concerns and perceptions among healthcare workers while facing the COVID-19 crisis and their correlation to burnout.

## 2. Materials and Methods

### 2.1. Procedure

The study was a cross-sectional study carried out at the Soroka University Medical Center in Israel, a large hospital in Israel’s southern peripheral area, which provides services to approximately one million people. The study received approval from the Hospital Ethics Committee (approval #0164-20-SOR). A total of 4000 staff members received a questionnaire via email from the Human Resources department on 9 July 2020. Participants gave their informed consent for inclusion in the study and were informed about anonymity, data protection, and privacy.

### 2.2. Participants

The research dataset consisted of 547 members of the hospital’s medical staff. After checking data from the hospital administration, we established that the characteristics of non-respondents (gender, age composition, and profession) were not statistically different compared to the study sample. Among the respondents, 75% were women, 88% were partnered, and 86% had children. The average age was 44 (SD = 11.6). Approximately half of the respondents were frontline workers and directly provided services for COVID-19 patients or treated COVID-19 patients. Table 1 shows a detailed description of the sample.

### 2.3. Instrument

The online survey included 30 questions that were pretested with eight employees working in a different hospital: 2 physicians, 2 nurses, 4 employees from other professions, e.g., administrative, social work, pharmacy), to ensure that they were relevant to hospital staff during the crisis. We conducted two online discussions using a Delphi method process. Based on the eight employees’ responses during the discussions, we arrived at a consensus regarding the finalized survey.

The questionnaire included nine sections, and each section comprised several questions/statements which the respondent was required to answer or rate, using a Likert scale, ranging from (1) not at all to (5) to a very great extent, plus the option “not relevant”:

1. Have you treated/are you directly treating COVID-19 patients at work?
2. Demographics—gender, age, profession, family status, children, was in quarantine, or has been infected with COVID-19 or been tested for COVID-19.
3. Self-assessment of personal coping with the pandemic—two statements. Reliability α = 0.88. Sample statement: “*On a personal level, I am dealing well with the pandemic.”*
4. Concerns regarding the COVID-19 crisis on an individual level—two statements. Reliability α = 0.55. Sample statement: “*I am concerned for my health.”*
5. Concerns regarding the COVID-19 crisis on a family level—three statements. Reliability α = 0.65. Sample statement: “*I am concerned for my family members’ health.”*
6. Concerns regarding the COVID-19 crisis on an organizational level—four statements. Reliability α = 0.82. Example statement: “*I am concerned about the shortage of beds to accommodate all patients.”*
7. Concerns regarding the COVID-19 crisis on a national level—four statements. Reliability α = 0.63. Example statement: “*I am concerned about infection rate in Israel.”*
8. Perceptions of crisis management—three statements. Reliability α = 0.73. A high score indicated more positive perceptions. Example statement: *“I support the way in which the country has handled the crisis so far.”*
9. Burnout—three statements examined the degree of emotional exhaustion. Reliability α = 0.82. A high score indicated a high level of burnout. Example statement: *“I feel burnt out from coping with the crisis.”*

### 2.4. Statistical Analysis

Data analysis was carried out using IBM SPSS Statistics 25.0 software. The exploratory data analysis demonstrated the normal distribution of the data, and parametric statistical tests were used. Reliability was examined using Cronbach’s alpha. Pearson correlations, *t*-tests for independent samples, one-way ANOVA, and multiple regression models were applied. The post hoc evaluation was calculated using Tukey’s method.

## 3. Results

The current study survey consisted of seven dimensions describing self-assessment of coping, concerns, perceptions, and burnout. Table 2 shows the means and standard deviations of the survey dimensions. The analysis shows the respondents’ deep concerns for families and apprehensions at a national level (for example, worries regarding the economic crisis accompanying the pandemic and increased spread of the virus). Respondents noted that they were coping well with the crisis while expressing negative perceptions of crisis management at the national level.

Table 3 shows the correlations between the survey dimensions. The analysis results reveal a positive association between the respondents’ concerns at the individual, family, organizational, and national levels and burnout. Negative correlations were found between self-assessment of personal coping with the crisis and burnout and between perceptions of crisis management and burnout.

Table 4 shows a means comparison between genders for survey dimensions. The analysis reveals gender differences across all dimensions, except for self-assessment of personal coping with the crisis and burnout. Women expressed more significant concerns than men at the individual, family, national, and organizational levels. However, women expressed slightly more positive perceptions of how the crisis was managed compared to men.

Table 5 shows variances between professions in the survey dimensions. A significant variance between professions was found in most of the tested dimensions, except for self-assessment of personal coping with the crisis, national-level concerns, and burnout. The analysis indicates that physicians reported a positive self-assessment of individual coping with the crisis and the lowest concerns across all levels. Additionally, nurses showed more positive perceptions towards crisis management than physicians.

An examination of variance between demographic variables and burnout found that respondents who provided medical care for COVID-19 patients reported higher burnout levels than those who did not (means = 3.29 and 2.86, respectively, F = 23.04, *p* < 0.001). Additionally, respondents who were not tested for COVID-19 but wanted to be tested reported higher levels of burnout than those who were tested and those who thought a test was not necessary (means = 3.29, 3.02, and 2.84, respectively, F = 6.27, *p* < 0.001).

Table 6 shows a multiple regression model for study variables and survey dimensions as predictors of burnout. The model presented in Table 6 included only variables which showed significant contribution to the prediction. The analysis of the assumed regression model shows that medical staff members’ low self-assessment of personal coping with the crisis, serious concerns at the organizational level, negative perceptions of crisis management, and providing medical care for COVID-19 patients are predictors of burnout. The variance explained by the final model was 18.6% of burnout (*p* < 0.001).

## 4. Discussion

The current study aimed to examine different aspects of the organizational atmosphere and their association with burnout during the global COVID-19 pandemic. The data analysis allowed us to conclude the significance of concerns and organizational perceptions of hospital staff in explaining burnout during the current crisis. Hospital staff expressed deep worries for families and apprehensions at a national level, noting that they were coping well with the crisis, and expressed moderate burnout levels, with women and nurses expressing higher burnout levels. Our findings coincide with recent research conducted at the Houston Methodist Hospital during the COVID-19 pandemic, which found that physicians and nurses had significant concerns for their families, hesitated to go home for fear of infecting family members, and experienced emotional exhaustion. This was especially evident among intensive care staff and frontline workers [20].

Prolonged high stress may lead to burnout [21], which affects the quality of medical care [22]. Previous studies of nurses revealed that factors such as management style and leadership, workload, and coping with the patients’ needs and their families were associated with stress and burnout [23,24,25]. Studies among physicians [26,27] found that occupational stressors (workload, long shifts, and work conflicts) contribute to an increase in burnout, which in turn leads to a decrease in the quality of medical care [28].

Nonetheless, in a recent study which used in-depth interviews of 14 physicians and nurses in Hubei Province in China, medical staff noted strong feelings of responsibility to provide good care to patients during the pandemic. Although they expressed worries of infecting relatives and about unforeseen risks or high workload, interviewees focused on their high level of responsibility and dedication to face the challenge and expressed feelings of self-efficacy while facing the global pandemic [29]. It seems that taking a significant role as medical staff during the current exceptional crisis created feelings of empowerment, and moderated burnout. In Israel, the policy of the Ministry of Health included referring COVID-19 patients to different hospitals in the country according to their capacity status, in order to keep a balance between hospitals and to prevent workload and staff burnout. Therefore, burnout levels were quite moderate.

At the same time, we found that having negative perceptions towards the crisis management and being a frontline worker directly exposed to COVID-19 patients was associated with burnout. Frontline medical staff working in emergency wards and intensive care units are at risk of suffering from stress and burnout, as they are working in a highly demanding environment [30], as in the COVID-19 crisis. In a recent cross-sectional study examining coping strategies and concerns of medical staff during the COVID-19 pandemic, researchers found that healthcare workers showed concern for their families and organizational aspects such as workers’ safety, availability of equipment and guidelines, and recognition of their efforts by hospital management. Additional concerns included workers’ expectations of receiving support from seniors and leaders, management monitoring of workers’ wellness, and proactively addressing safety concerns [31]. In a study conducted at the beginning of the pandemic in the United States, medical staff noted that they did not expect leaders to provide solutions for every issue that arose during the crisis. However, they noted the importance of acknowledgment of their needs, and considering their expertise as an important part of the organizational and systemic preparedness strategies for confronting the crisis [32]. Managers have a vital role in addressing of medical staff relating to COVID-19 by providing supportive organizational plans and maintaining a safe work environment that assists healthcare workers facing the unique challenges imposed by the COVID-19 pandemic [33].

Healthcare organizations must understand the main stressors during COVID-19 and the factors that can mitigate the negative influence of the pandemic on the mental health of medical staff. There is an urgent need to develop plans and strategies to address the root causes of stressors and concerns and maintain efficient and rapid communication with health workers, transparency, and support.

### Limitations

The current study has several limitations. A small sample of medical staff from a single hospital answered the survey. A large-scale sample comparing different hospitals is recommended to broaden the conclusions. Due to the COVID-19 situation, it was challenging to achieve high participation rates. Follow-up evaluation of concerns and burnout, and of supportive services provided to medical staff during the COVID-19 crisis, was not within the scope of this study. In addition, the current study did not use tools to assess psychological measures such as depression and anxiety.

## 5. Conclusions and Recommendations

The current study results expose the main concerns and perceptions of frontline medical staff during the COVID-19 crisis and present issues that require immediate organizational attention to increase the resilience of medical staff in times of global crisis. Recent research suggested three dimensions of moderators which may reduce adverse mental outcomes such as emotional exhaustion and burnout among healthcare workers: organizational moderators such as occupational safety and health management, institutional moderators such as government programs that aim to provide financial and psychological support to workers, and individual moderators such as social support and wellbeing [34].

Various steps are required to improve health organizations’ preparedness to confront the continuous COVID-19 pandemic and future health crises. These include developing a supportive organizational culture, providing a psychosocial support plan for frontline workers, ensuring their safety and health while they provide medical care for patients, and preventing burnout. These steps will help secure the human resources critical to cope with the global health crisis.

## Figures and Tables

**Table 1:** Demographic characteristics of the sample (*n* = 547).

Variable		N	%
Gender	Male	137	25%
	Female	410	75%
Marital status	In a relationship	446	82%
	Not in a relationship	101	18%
Children	None	73	13%
	Aged 0–10	219	40%
	Aged >10	249	46%
Provided services/treated coronavirus patients as part of their job	Yes	249	46%
	No	298	54%
Quarantined	Yes	66	12%
	No	481	88%
COVID-19 test	Tested	249	46%
	Was not required	233	42%
	No, although a test was requested/required	65	12%
Role	Physician	91	17%
	Nurse	177	32%
	Other *	279	51%

* Other—administrative and housekeeping, computing, auxiliary staff, laboratory.

**Table 2:** Means and standard deviations of the survey dimensions.

Dimension	Mean	SD
Personal coping with the crisis	3.94	0.80
Individual-level concerns	3.78	0.92
Family-level concerns	4.22	0.76
National-level concerns	4.19	0.81
Organizational-level concerns	3.57	0.99
Perceptions of crisis management	2.48	0.91
Burnout	2.98	0.94

**Table 3:** Pearson correlations between the survey dimensions (*n* = 547).

Dimension	Individual-Level Concerns	Family-Level Concerns	National-Level Concerns	Organizational-Level Concerns	Perceptions of Crisis Management	Burnout
Personal coping with the crisis	−0.12 *	−0.20 **	−0.21 **	−0.23 **	0.15 **	−0.23 **
Individual-level concerns		0.63 **	0.46 **	0.51 **	0.19 **	0.12 **
Family-level concerns			0.49 **	0.62 **	0.06	0.26 **
National-level concerns				0.67 **	0.06	0.21 **
Organizational-level concerns					0.10 *	0.29 *
Perceptions of crisis management						−0.13 *

* *p* < 0.05, ** *p* < 0.001.

**Table 4:** Means comparison between genders for survey dimensions.

Dimension	Women (*n* = 410)		Men (*n* = 137)		t	*p*
	M	SD	M	SD		
Personal coping with the crisis	3.93	0.78	3.98	0.86	0.69	NS
Individual-level concerns	3.87	0.89	3.49	0.94	−4.23	0.000
Family-level concerns	4.30	0.71	3.97	0.83	−4.55	0.000
National-level concerns	4.25	0.78	3.99	0.89	−3.24	0.000
Organizational-level concerns	3.66	0.98	3.29	0.99	−3.85	0.000
Perceptions of crisis management	2.55	0.87	2.27	0.98	−3.09	0.000
Burnout	3.02	0.94	2.86	0.94	−1.65	NS

t—t test statistic, *p*-probability.

**Table 5:** Variances between the professions in the survey dimensions.

Dimension	Role	Mean	SD	95% Confidence Interval for Mean		F	*p*
				Upper Bound	Lower Bound		
Individual coping with the crisis	Physicians	4	0.09	4.10	3.76	0.61	NS
	Nurses	3.93	0.06	4.12	3.88		
	Other	3.91	0.05	4.01	3.82		
Individual-level concerns	Physicians	3.25	0.09	3.44	3.07	19.14	0.001
	Nurses	3.86	0.07	4.00	3.73		
	Other	3.90	0.05	4.01	3.80		
Family-level concerns	Physicians	3.90	0.08	4.06	3.75	10.22	0.001
	Nurses	4.31	0.06	4.42	4.19		
	Other	4.28	0.05	4.37	4.19		
National-level concerns	Physicians	4.04	0.09	4.21	3.88	2.05	NS
	Nurses	4.25	0.06	4.38	4.13		
	Other	4.21	0.05	4.31	4.11		
Organizational-level concerns	Physicians	3.29	0.10	3.49	3.08	5.38	0.004
	Nurses	3.70	0.07	3.85	3.56		
	Other	3.59	0.06	3.71	3.48		
Perceptions of crisis management	Physicians	2.05	0.09	2.23	1.86	13.56	0.001
	Nurses	2.49	0.07	2.62	2.36		
	Other	2.61	0.05	2.72	2.50		
Burnout	Physicians	2.98	0.10	3.18	2.79	2.08	NS
	Nurses	3.10	0.07	3.24	2.96		
	Other	2.92	0.06	3.03	2.80		

F-one-way anova statistic, *p*-probability. NS–not statictically significant.

**Table 6:** Multiple regression model for study variables and survey dimensions as predictors of burnout.

Dimension/Variable	B	Beta	*p*
Personal coping with the crisis	−0.16	−0.14	0.000
Organizational-level concerns	0.21	0.22	0.000
Perceptions toward crisis management	−0.12	−0.12	0.003
Treated COVID-19 patients	−0.48	−0.23	0.000

B-unstandardized beta, *p*-probability.

## Data Availability

The data that support the findings of this study are available from the corresponding author.
